# Highly selective olefin-assisted palladium-catalyzed oxidative carbocyclization *via* remote olefin insertion†
†Electronic supplementary information (ESI) available. See DOI: 10.1039/c6sc02660e



**DOI:** 10.1039/c6sc02660e

**Published:** 2016-09-15

**Authors:** Youai Qiu, Bin Yang, Can Zhu, Jan-E. Bäckvall

**Affiliations:** a Department of Organic Chemistry , Arrhenius Laboratory , Stockholm University , SE-106 91 , Stockholm , Sweden . Email: jeb@organ.su.se

## Abstract

A highly selective olefin-assisted palladium-catalyzed oxidative carbocyclization *via* remote olefin insertion to afford cyclohexenes has been developed. It was shown that the assisting olefin moiety was indispensable for the formation of the cyclohexene product. Furthermore, preliminary studies on chiral anion-induced asymmetrical carbocyclization–borylation of enallenes have been carried out.

## Abstract 2

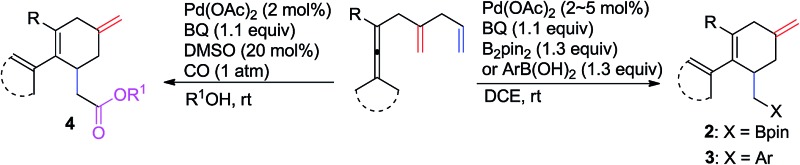
An olefin-assisted palladium-catalyzed oxidative carbocyclization of enallenes to afford cyclohexene skeletons involving ligand exchange of olefins has been established.

The development of modern methodologies for the efficient synthesis of carbocycles is of central importance in modern organic chemistry,^1^ given the fact that carbocycles are basic units in pharmacologically active skeletons, as well as in natural products.^2^ Among these methodologies, transition metal-catalyzed carbocyclizations with the involvement of π-bonds have emerged as an effective strategy for the preparation of carbocycles,^3,4^ considering that the π-bond moiety would not only perform as the assisting group for the formation of the carbon–metal (C–M) bond, but also as the building block for the subsequent carbocyclization. In this way, an atom-economical transformation can be achieved.

We recently reported on an olefin-directed palladium-catalyzed oxidative carbocyclization–borylation of allenes to cyclobutenes (Scheme 1a).^5^ In this reaction, the coordination of the olefin in ***Int*-A** triggers the allene attack on palladium,^6^ which results in the formation of ***Int*-B**. Subsequent olefin insertion to form a cyclobutene intermediate ***Int*-C**, followed by transmetallation and reductive elimination afforded the borylated cyclobutene derivatives **A**.

**Scheme 1:**
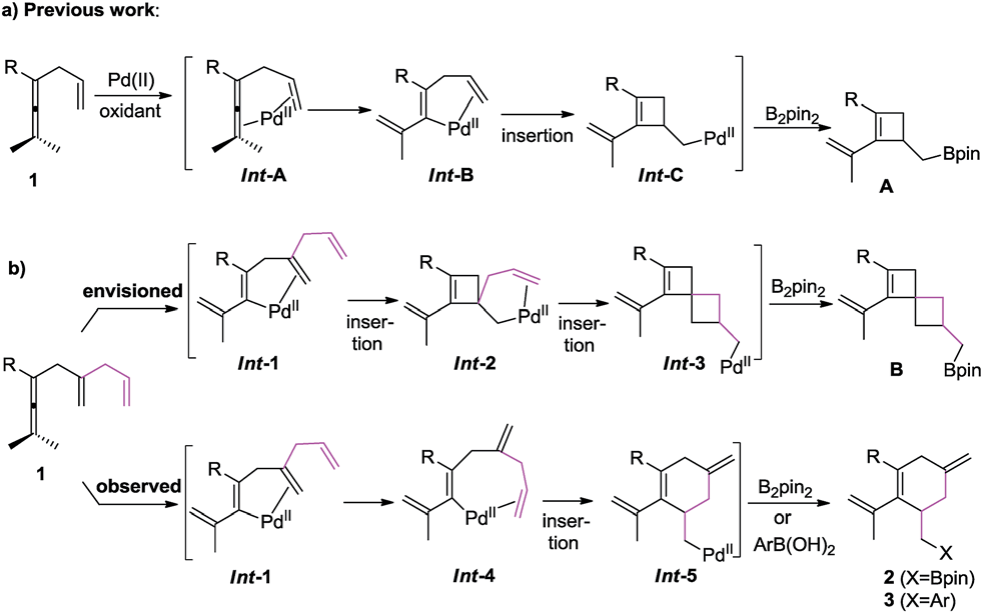
Previous reports and this approach.

On the basis of these observations, we were particularly interested in the involvement of an additional double bond in a carbocyclization (Scheme 1b). We envisioned that the olefin insertion of intermediate ***Int*-1** could lead to intermediate ***Int*-2**, and that coordination of the additional olefin to palladium would lead to a second carbocyclization to form spirocyclic intermediate ***Int*-3**, which on reaction with B_2_pin_2_ would give **B**. Alternatively, ***Int*-1** may undergo ligand exchange and olefin insertion to give ***Int*-5**, which can be quenched by either B_2_pin_2_ or ArB(OH)_2_ to give either **2** or **3**, respectively (Scheme 1b).

Based on this concept, we initially chose a readily accessible 3,4-dienoate **1a** as the standard substrate. When **1a** bearing an extra olefin was treated with Pd(OAc)_2_ (5 mol%), B_2_pin_2_ (1.3 equiv.), and BQ (*p*-benzoquinone) (1.1 equiv.) in THF at room temperature for 12 h, the envisioned spirocyclic product **B** was not observed (Scheme 2). Interestingly, the cyclohexene product **2a** was obtained in 76% yield instead. It is obvious that intermediate ***Int*-2** was not formed from intermediate ***Int*-1** *via* olefin insertion as we had envisioned, but rather the ligand exchange, from proximal olefin to remote olefin occurred in ***Int*-1** to produce ***Int*-4**. Subsequent olefin insertion^7^ to give cyclic intermediate ***Int*-5** followed by B_2_pin_2_ quenching would produce **2a** (Scheme 1b). During this transformation, the proximal olefin is acting as the assisting group for the generation of palladium intermediate ***Int*-1**, while the remote olefin participates in the carbocyclization (Scheme 1c). To the best of our knowledge,^8–10^ the formation of six-membered rings in palladium-catalyzed oxidative carbocyclization of enallenes *via* olefin exchange has been rarely reported.^11^

**Scheme 2:**
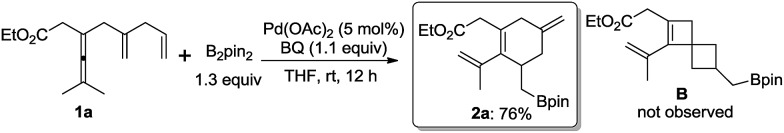
Palladium-catalyzed oxidative carbocyclization–borylation of enallene **1a**.

To demonstrate the necessity of the assisting olefin group, we first investigated comparative experiments with enallenes lacking the additional olefin (Scheme 3). When substrate **1f** with an assisting olefin was allowed to react under the same reaction conditions as those in Scheme 2, the cyclohexene product **2f** was formed in 70% yield (Scheme 3a). However, when substrate **1fb**, lacking the additional double bond, was subjected to the reaction conditions of Scheme 2 the corresponding six-membered ring product **2fb** was not formed; instead **1fb** was recovered in 88% (Scheme 3b). Importantly, we also observed the exclusive formation of **2h** in 68% yield from substrate **1h** (Scheme 3c). We also examined the reaction of a malonate-tethered substrate **1l**, but the envisioned product **2l** was not observed (Scheme 3d). These comparative experiments indicate that the assisting olefin of the substrate is an indispensable group for the formation of the palladium intermediate ***Int*-4**.

**Scheme 3:**
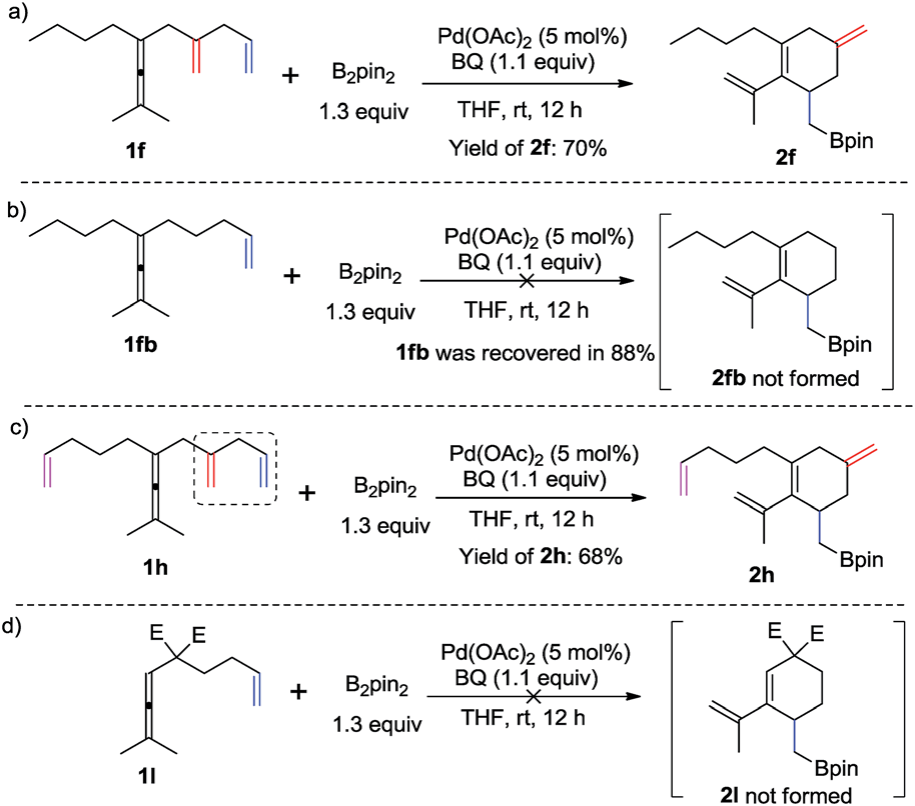
Comparative experiments.

With these inspiring results in hand, we began to optimize the reaction conditions (for details, see the ESI, Table S1†). Solvent screening showed that 1,2-dichloroethane was the best solvent, in which the yield of **2a** was 91% (Table S1,† entry 7). Other solvents such as THF, MeCN, and 1,4-dioxane also gave good yields (Table S1,† entries 1, 5, and 6). When the amount of BQ was increased to 1.5 equivalents, the yield of **2a** was decreased to 78% (Table S1,† entry 8). The use of 2,6-dimethyl-BQ instead of BQ, gave a lower yield (Table S1,† entry 9). Catalyst screening showed that Pd(TFA)_2_ (TFA = trifluoroacetate) produced the corresponding cyclohexene derivative in only 36% yield together with 34% starting material recovered (Table S1,† entry 10).

The substrate scope for the formation of cyclohexene boron compounds **2** ^12,13^ was then studied under the optimized reaction conditions (Scheme 4): in addition to methyl substituents on the enallene moiety, cyclobutylidene, cyclopentylidene, and cyclohexylidene enallenes (**1b**, **1c**, and **1d**) also gave the corresponding products **2b**, **2c**, and **2d** in good yields. To our delight, enallenes with functional groups, such as free hydroxyl in **1e** and imide in **lk**, furnished cyclohexene derivatives **2e** and **2k** in 83% and 76% yield. Furthermore, the reaction tolerates R to be different alkyl groups in this reaction, *e.g. n*-butyl (**1f**), or benzyl (**1g**).^14^ It is worth noting that the product **2h** was exclusively obtained in 84% yield. Finally, the reaction of a dissymmetric allene **1i**, bearing Me and phenyl, or **1j**, bearing Me and i-Pr, afforded **2i** in 86% yield, and **2j** and **2j′** in 60% yield, respectively. The ratio of **2j** and **2j′** was 1 : 2 due to the selective C–H bond cleavage, which occurred during allene attack forming ***Int*-1** (see Scheme 1b). Notably, the reaction could be easily extended to a scale of 4.5 mmol of **1a** (1.053 g) to afford the corresponding cyclohexene compound **2a** (1.551 g, 90% yield).

**Scheme 4:**
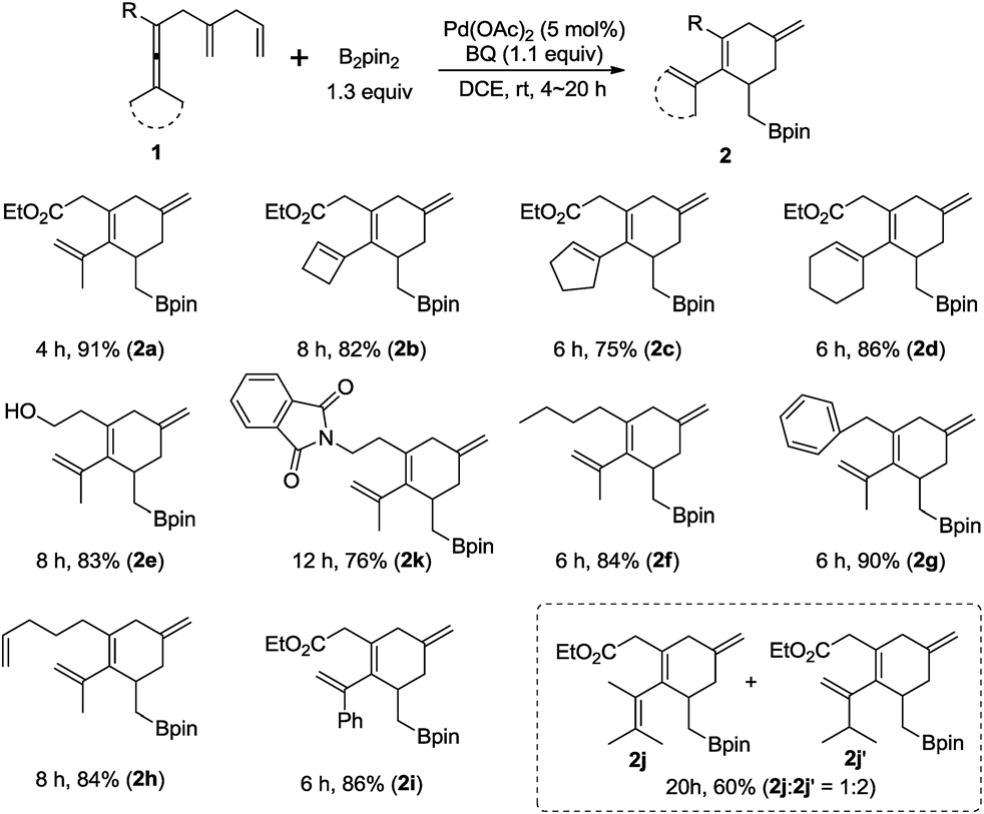
Scope of palladium-catalyzed oxidative borylating carbocyclization. The reaction was conducted in DCE at room temperature using **1** (0.2 mmol), B_2_pin_2_ (1.3 equiv.), and BQ (1.1 equiv.) in the presence of Pd(OAc)_2_ (5 mol%).

After realization of the borylative carbocyclization, we next turned our attention to the arylating carbocyclization of enallenes. We were pleased to find that the arylative products^15^ could be obtained in good yields with a catalyst loading of 2 mol% Pd(OAc)_2_ (Scheme 5). The reaction of substrates with two methyls, cyclopentylidene, and cyclohexylidene, afforded the corresponding product **3a**, **3c**, and **3d** in good yields. Interestingly, the substrate containing a free hydroxyl group could also be employed. Different alkyl substituents on the starting materials, such as *n*-butyl, benzyl, and 4-pentenyl groups were tolerated (**3f–h**). We also examined the scope of arylboronic acids, and electron-donating substituents such as 3-Me, and 3-MeO reacted smoothly under the standard conditions in good yields. Notably, the procedure tolerates a range of additional functional groups on the arylboronic acid, including bromo (**3ad**), vinyl (**3ae**), formyl (**3af**), and acetyl (**3ag**) groups, which is useful for further functionalization. Finally, it is worth noting that 2-naphthylboronic acid and 1-naphthylboronic acid also worked well, affording **3ah** and **3ai** in 84% and 55% yield, respectively.

**Scheme 5:**
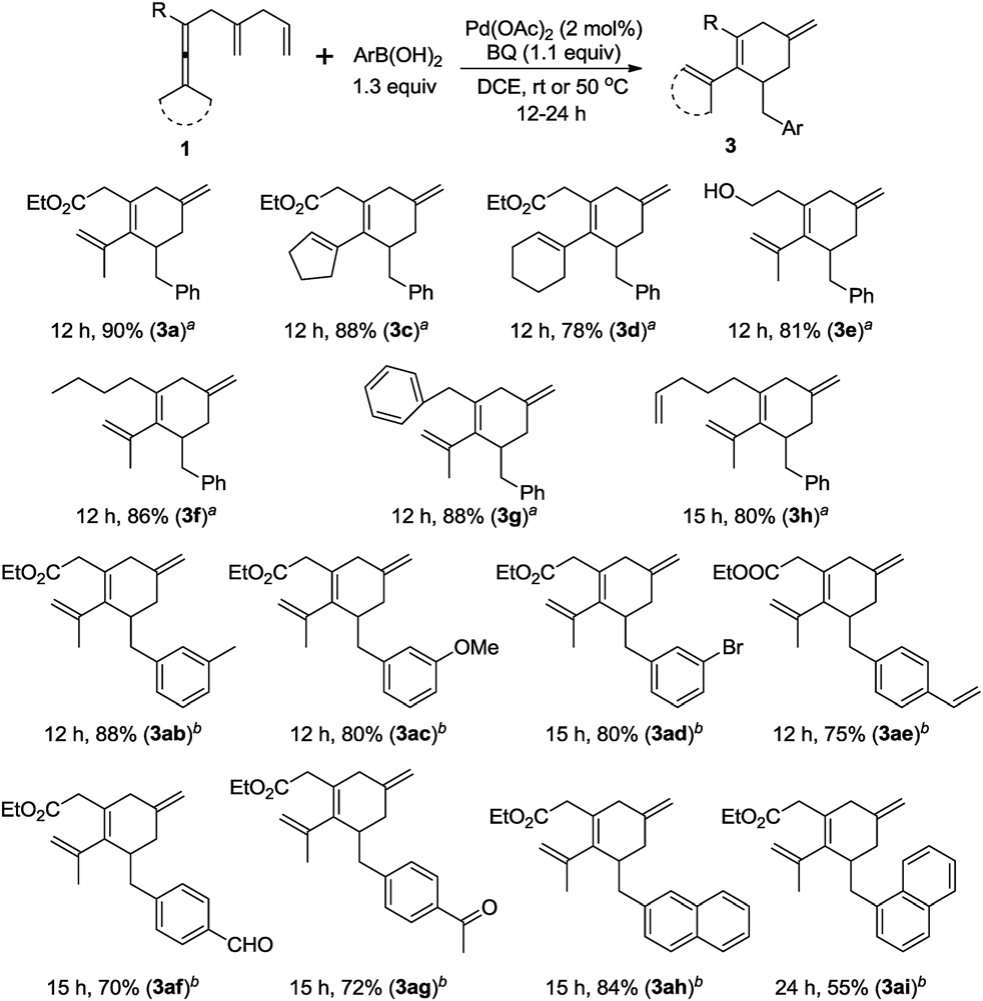
Scope of the palladium-catalyzed oxidative arylating carbocyclization. ^*a*^ The reaction was conducted in DCE at room temperature using **1** (0.2 mmol), ArB(OH)_2_ (1.3 equiv.), and BQ (1.1 equiv.) in the presence of Pd(OAc)_2_ (2 mol%). ^*b*^ The reaction was conducted at 50 °C.

Interestingly, this new olefin-assisting strategy could also be applied to an oxidative carbonylating carbocyclization^9c,f,g^ for the preparation of cyclohexene esters (Scheme 6).^16^ When the substrate **1a** was treated with Pd(OAc)_2_ (2 mol%), and BQ (1.1 equiv.) under carbon monoxide (1 atm) in methanol at room temperature for 12 h the carbonylation product **4a** was formed in 82% yield (Scheme 6). Under the optimal reaction conditions, cyclopentylidene and cyclohexylidene substrates **1c** and **1d** afforded **4c** and **4d**, respectively in good yields. The substrate bearing the free hydroxyl group (**1e**) also worked well. Different allenes with alkyl substituents such as *n*-butyl, benzyl, and 4-pentenyl group, were also tested and worked well in this reaction. The reaction of a dissymmetric allene **1i**, bearing Me and phenyl, afforded **4i** in 70% yield. Finally, the scope of the alcohol partners in the carbocyclization carbonylation reaction was explored, and in addition to MeOH, ethanol and isopropanol were shown to react smoothly to provide the desired esters in good yield.

**Scheme 6:**
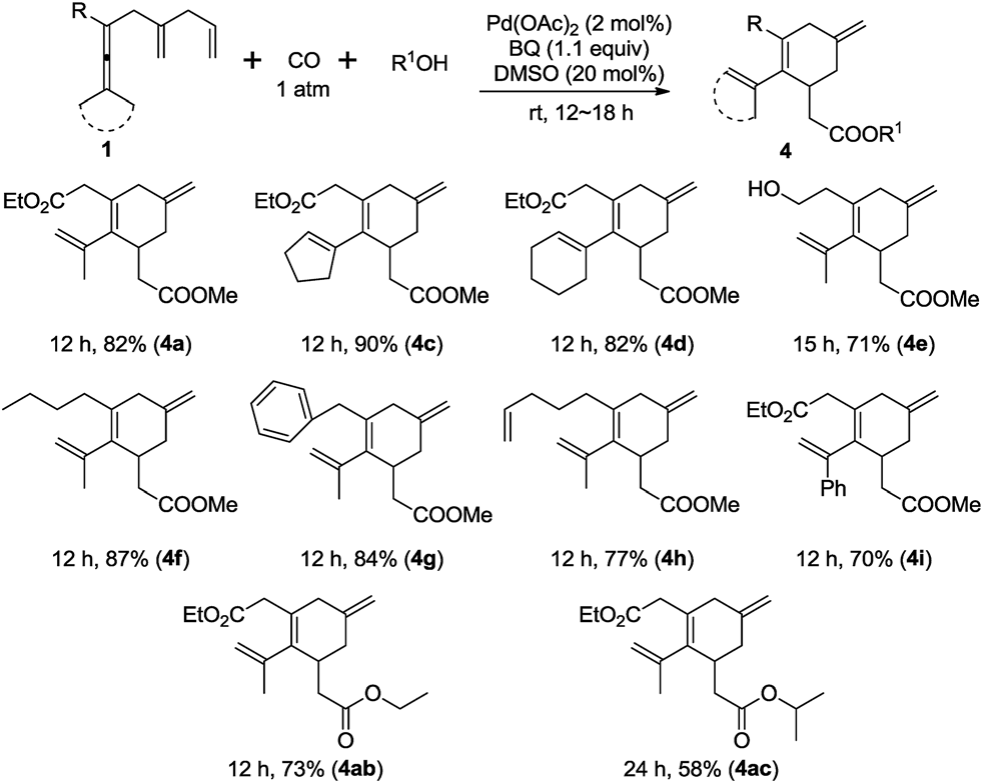
Scope of palladium-catalyzed oxidative carbonylating carbocyclization. The reaction was conducted in R^1^OH using **1** (0.2 mmol), DMSO (20 mol%), and BQ (1.1 equiv.) in the presence of Pd(OAc)_2_ (2 mol%).

The biomimetic approach with the use of electron-transfer mediators (ETMs) is known to decrease the kinetic barrier for the reoxidation.^17^ In this aerobic approach the high kinetic barrier will be divided into several smaller units, and catalytic amounts of oxidant (BQ) would be enough to realize these transformations. When the reaction of **1a** was treated with B_2_pin_2_ (1.3 equiv.), BQ (20 mol%), Pd(OAc)_2_ (5 mol%), and cobalt(salophen) (5 mol%) in the presence of O_2_ (1 atm), borylated product **2a** was obtained in 89% yield. Phenylated product **3a** was provided in 86% yield when PhB(OH)_2_ was used in place of B_2_pin_2_ (Scheme 7).

**Scheme 7:**
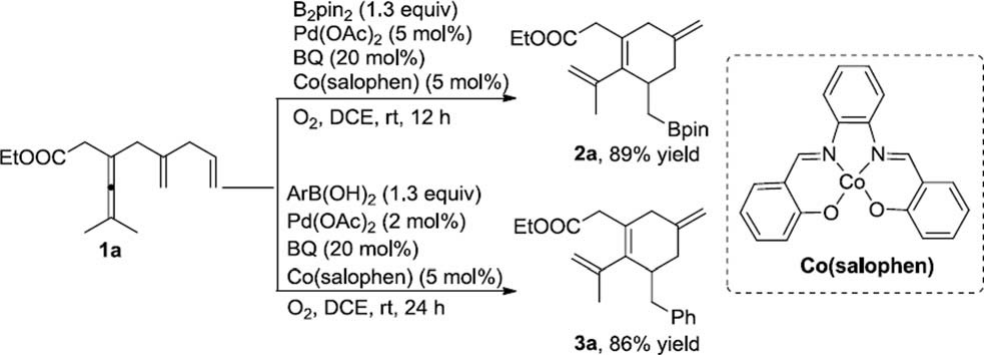
Palladium-catalyzed oxidative carbocyclization under biomimetic conditions.

Preliminary attempt to develop an enantioselective carbocyclization–borylation of enallenes revealed that a reasonably good er value (83 : 17) was observed in the presence of catalytic amounts of Pd(OAc)_2_ and biphenol-type phosphoric acid **C** ^9*d*^ while poor enantiocontrol (55 : 45 er) was obtained with VAPOL phosphoric acid (Scheme 8).^18,19^

**Scheme 8:**
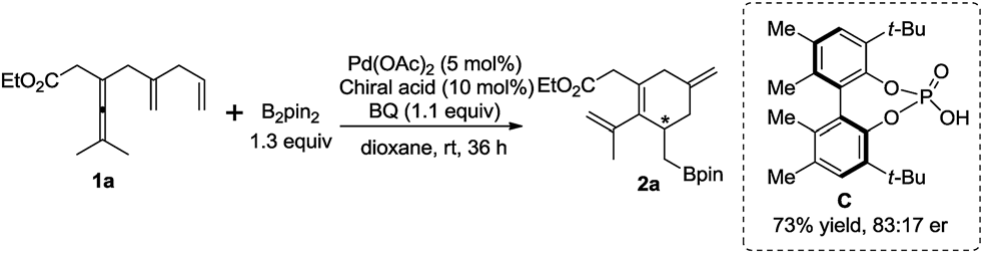
Preliminary results of the asymmetric carbocyclization–borylation.

Based on the experiments in Scheme 3 and the reaction outcome, a possible mechanism for the olefin-assisted palladium-catalyzed oxidative carbocyclization of enallenes *via* remote olefin insertion is given in Scheme 9. The reaction of palladium with enallene **1** bearing the assisting olefin forms vinylpalladium intermediate ***Int*-1** *via* allene attack involving allenic C–H bond cleavage, which is promoted by the coordination of allene and the assisting olefin to Pd(ii).^5,6^ Then, the vinylpalladium intermediate ***Int*-4** would be generated from ***Int*-1** *via* ligand exchange (from proximal olefin to remote olefin), instead of a direct olefin insertion to form cyclobutene complex ***Int*-2**.^5^ Intermediate ***Int*-4** would undergo a remote olefin insertion to give cyclic intermediate ***Int*-5**. Subsequent transmetallation of ***Int*-5** with B_2_pin_2_ or arylboronic acid, followed by reductive elimination would give the target cyclohexene derivatives **2** or **3**. Under CO pressure in alcohol, ***Int*-5** can undergo an alkoxy-carbonylation to provide product **4**.

**Scheme 9:**
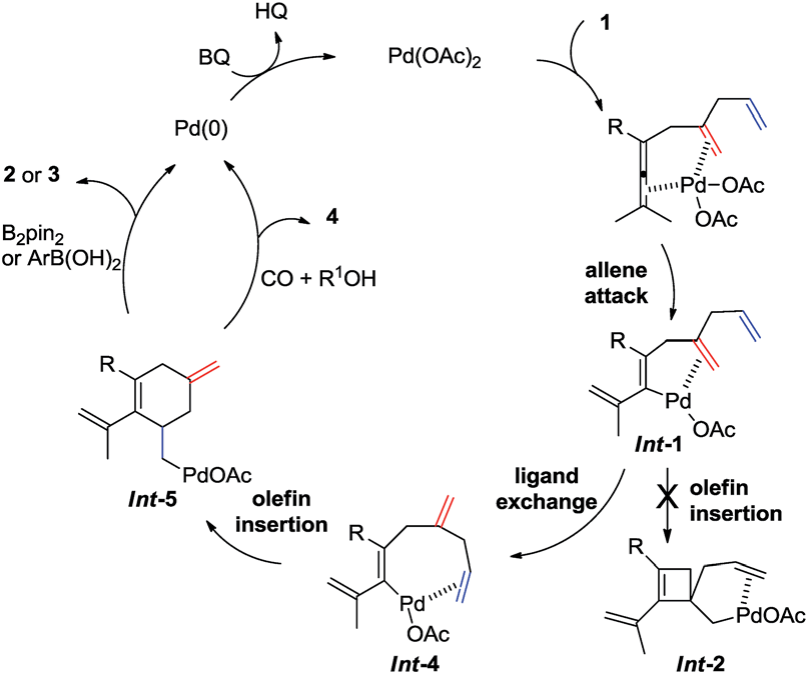
Proposed mechanisms.

## Conclusions

In conclusion, we have developed a highly selective olefin-assisted palladium-catalyzed oxidative carbocyclization of enallenes *via* remote olefin insertion for the selective formation of the cyclohexene skeleton. It was demonstrated that the assisting olefin moiety is essential for the formation of the cyclohexene derivatives. These reactions all show a broad substrate scope and good tolerance for various functional groups, and the catalyst loading could be decreased to 2 mol% in the arylative and carbonylative reactions with good to excellent yields. The biphenol-type chiral phosphoric acid was used in preliminary experiments of enantioselective carbocyclization–borylation of enallene. Further studies on the scope, synthetic application, and asymmetric variants of these reactions are currently carried out in our laboratory.
